# Supplementary material for: Genetic Markers for Thrombophilia and Cardiovascular Disease Associated with Multiple Sclerosis
Source: Biomedicines. 2022 Oct 21;10(10):2665. doi: 10.3390/biomedicines10102665 (PMC9599167; doi:10.3390/biomedicines10102665)
Supplement: Supplementary file 1 [file biomedicines-10-02665-s001.zip › biomedicines-1968214-supplementary.pdf]

Supplementary Table S1: Demographic and laboratory findings of healthy controls (HC) for thrombophilia risk factors

No	Age	Gender	Factor V Leiden (R506Q)	Factor V R2 (H1299R)	Prothrombin (20210G>A)	Factor XIII V34L	beta-Fibrinogen -455 G>A	Human Platelet Antigen 1 (HPA-1) a/b
HC-1	39	F	_	_	_	_	_	a/a
HC-2	26	M	Heterozygous	_	_	Heterozygous	Heterozygous	a/a
HC-3	30	F	_	_	_	_	_	a/b
HC-4	43	M	_	_	_	_	_	a/a
HC-5	55	M	_	_	_	Heterozygous	Heterozygous	a/b
HC-6	51	F	_	_	_	Heterozygous	_	a/a
HC-7	45	F	_	_	_	_	_	a/b
HC-8	34	F	_	_	_	Heterozygous	Heterozygous	a/a
HC-9	30	M	_	_	_	Heterozygous	Heterozygous	a/a
HC-10	47	M	_	Heterozygous	_	Heterozygous	_	a/a
HC-11	44	F	Heterozygous	_	_	_	_	a/b
HC-12	30	F	_	_	_	Heterozygous	_	a/b
HC-13	50	M	Heterozygous	_	_	Homozygous	Heterozygous	a/a
HC-14	54	F	_	_	_	Heterozygous	_	a/b
HC-15	54	F	_	_	_	_	_	b/b
HC-16	26	F	_	_	_	_	_	a/a
HC-17	46	F	_	_	_	Heterozygous	Heterozygous	a/a
HC-18	21	F	_	Heterozygous	_	Heterozygous	_	a/a
HC-19	39	M	_	_	_	_	_	a/b
HC-20	50	F	_	Heterozygous	_	_	_	a/a
HC-21	36	M	_	_	_	_	Heterozygous	a/a
HC-22	44	F	_	_	_	_	Heterozygous	a/a
HC-23	42	F	_	_	_	Heterozygous	_	a/a
HC-24	51	F	_	Heterozygous	_	Heterozygous	Heterozygous	a/a
HC-25	31	M	_	_	_	_	_	a/a

Supplementary Table S2: Demographic and laboratory findings of healthy controls (HC) for cardiovascular risk factors

**No**	**Age**	**Gender**	**Plasminogen activator inhibitor 1 (PAI-1) 4G/5G**	**Methylenetetrahydrofolate reductase (MTHFR) 677C>T**	**Methylenetetrahydrofolate reductase (MTHFR) 1298A>C**	**Angiotensin-Converting Enzyme (ACE) I/D**	**Apolipoprotein B (Apo) B R3500Q**
HC-1	39	F	4G/4G	_	Homozygous	I/D	_
HC-2	26	M	4G/4G	Heterozygous	_	D/D	_
HC-3	30	F	4G/5G	_	Heterozygous	D/D	_
HC-4	43	M	4G/4G	_	Heterozygous	I/I	_
HC-5	55	M	4G/4G	Homozygous	_	D/D	_
HC-6	51	F	4G/5G	_	_	I/D	_
HC-7	45	F	4G/5G	Heterozygous	Heterozygous	I/D	_
HC-8	34	F	5G/5G	Heterozygous	_	D/D	_
HC-9	30	M	4G/5G	_	Heterozygous	I/D	_
HC-10	47	M	4G/4G	_	Heterozygous	D/D	_
HC-11	44	F	4G/5G	Heterozygous	_	I/D	_
HC-12	30	F	4G/5G	Homozygous	_	D/D	_
HC-13	50	M	4G/5G	Heterozygous	Heterozygous	D/D	_
HC-14	54	F	4G/5G	_	_	D/D	_
HC-15	54	F	5G/5G	_	Homozygous	I/I	_
HC-16	26	F	4G/4G	_	Homozygous	I/I	_
HC-17	46	F	4G/5G	Heterozygous	Heterozygous	I/D	_
HC-18	21	F	4G/5G	_	Heterozygous	I/D	_
HC-19	39	M	4G/5G	Homozygous	_	D/D	_
HC-20	50	F	4G/4G	Heterozygous	Heterozygous	I/D	_
HC-21	36	M	4G/5G	_	_	D/D	_
HC-22	44	F	5G/5G	Heterozygous	Heterozygous	D/D	_
HC-23	42	F	4G/4G	Heterozygous	Heterozygous	I/D	_
HC-24	51	F	4G/5G	_	Heterozygous	I/I	_
HC-25	31	M	4G/5G	Homozygous	_	D/D	_

Supplementary Table S3: Demographic, clinical, and laboratory findings of patients with multiple sclerosis (MS)

No	Age	Gender	Disease course	Disease duration	EDSS	MSSS	IgG antibodies against coagulation components
MS-1	56	M	RRMS	33	8.0	8.47	anti-FXa
MS-2	80	M	SPMS	28	6.0	4.54	anti-plasmin
MS-3	71	F	SPMS	29	5.0	3.01	anti-FXII, anti-protein C
MS-4	57	F	RRMS	23	4.0	2.78	anti-FXa
MS-5	47	F	RRMS	27	1.0	0.22	anti-FXII
MS-6	59	F	RRMS	30	2.5	1.19	anti-FVIIa
MS-7	50	M	RRMS	17	2.5	1.77	anti-plasmin
MS-8	58	M	SPMS	24	6.0	5.03	anti-plasmin
MS-9	56	M	RRMS	33	5.5	3.50	anti-FVIIa, anti-plasmin
MS-10	47	F	SPMS	21	4.0	2.97	anti-plasmin, anti-FXa, anti-FXII
MS-11	65	F	RRMS	25	3.0	1.56	anti-plasmin, anti-FXa
MS-12	46	F	RRMS	19	3.5	2.50	anti-FXII
MS-13	67	M	RRMS	22	4.0	2.82	anti-FXII
MS-14	36	F	RRMS	19	2.0	1.00	anti-FVIIa
MS-15	74	M	SPMS	20	6.5	6.43	anti-plasmin
MS-16	71	F	PPMS	20	5.5	4.30	anti-FVIIa, anti-plasmin
MS-17	40	F	RRMS	19	4.0	3.19	anti-FVIIa
MS-18	51	F	RRMS	21	5.5	4.21	anti-prothrombin
MS-19	60	F	RRMS	19	5.5	4.49	anti-plasmin
MS-20	63	M	SPMS	18	7.5	8.23	anti-plasmin
MS-21	80	M	RRMS	25	5.5	3.74	anti-plasmin
MS-22	45	F	RRMS	17	3.0	2.30	anti-plasmin, anti-FXa, anti-thrombin
MS-23	47	M	RRMS	17	4.0	3.65	anti-FXII
MS-24	54	M	RRMS	13	4.0	4.38	anti-FVIIa, anti-prothrombin
MS-25	44	F	RRMS	17	3.0	2.30	anti-thrombin

Supplementary Table S3: Demographic, clinical, and laboratory findings of patients with multiple sclerosis (MS)

No	Age	Gender	Disease course	Disease duration	EDSS	MSSS	IgG antibodies against coagulation components
MS-26	33	M	RRMS	13	3.0	3.05	anti-plasmin, anti-prothrombin, anti-FXII, anti-protein C
MS-27	52	M	RRMS	12	2.5	2.64	anti-FVIIa
MS-28	30	F	RRMS	11	3.0	3.46	anti-FXa
MS-29	52	F	RRMS	12	2.5	2.64	anti-prothrombin
MS-30	50	F	SPMS	12	5.5	6.03	anti-Fxa
MS-31	62	M	SPMS	10	7.0	8.92	anti-FXII
MS-32	60	F	RRMS	10	2.5	3.10	anti-protein C
MS-33	39	F	RRMS	10	3.0	3.79	anti-prothrombin
MS-34	45	F	RRMS	6	4.0	6.61	anti-plasmin
MS-35	29	F	RRMS	6	2.5	4.55	anti-FVIIa
MS-36	45	F	RRMS	9	5.0	6.50	anti-thrombin
MS-37	30	M	RRMS	0	1.0	_	anti-prothrombin
MS-38	43	F	RRMS	5	3.0	5.79	anti-FXa
MS-39	37	M	RRMS	7	1.5	2.10	anti-thrombin
MS-40	39	F	RRMS	2	3.0	6.81	anti-FXa, anti-protein C
MS-41	33	F	RRMS	11	2.5	2.82	anti-FXa, anti-protein C
MS-42	46	F	RRMS	15	2.0	1.64	anti-FVIIa
MS-43	46	M	RRMS	4	1.0	1.45	anti-FVIIa
MS-44	34	M	RRMS	3	2.0	4.82	anti-FXII
MS-45	36	M	RRMS	11	3.5	4.21	anti-FVIIa
MS-46	30	F	RRMS	4	3.0	6.24	anti-thrombin
MS-47	23	M	RRMS	4	2.5	5.41	anti-prothrombin
MS-48	57	F	RRMS	12	8.0	9.43	anti-plasmin, anti-FXa, anti-protein C, anti-thrombin

Supplementary Table S4: Laboratory findings of patients with multiple sclerosis (MS) for thrombophilia risk factors

No	Factor V Leiden (R506Q)	Factor V R2 (H1299R)	Prothrombin (20210G>A)	Factor XIII V34L	beta-Fibrinogen -455 G>A	Human Platelet Antigen 1 (HPA-1) a/b
MS-1	_	_	_	_	_	a/b
MS-2	_	_	_	_	_	a/b
MS-3	_	_	_	_	Heterozygous	a/b
MS-4	_	_	_	Heterozygous	_	a/b
MS-5	_	Heterozygous	_	_	Heterozygous	a/a
MS-6	_	_	_	_	_	a/a
MS-7	Heterozygous	_	_	_	_	a/a
MS-8	_	_	_	_	_	a/a
MS-9	_	_	_	Heterozygous	Heterozygous	a/a
MS-10	Heterozygous	_	_	_	_	a/a
MS-11	_	_	_	Heterozygous	_	a/a
MS-12	Heterozygous	_	_	_	_	a/a
MS-13	_	_	_	_	Heterozygous	a/a
MS-14	_	_	_	_	Heterozygous	a/a
MS-15	_	_	_	Heterozygous	Heterozygous	a/b
MS-16	_	_	_	_	Heterozygous	a/a
MS-17	_	_	_	_	Heterozygous	a/a
MS-18	_	Heterozygous	_	_	Heterozygous	a/a
MS-19	_	Heterozygous	_	_	Heterozygous	a/b
MS-20	_	_	_	Heterozygous	Heterozygous	a/a
MS-21	_	_	_	_	_	a/a
MS-22	Heterozygous	_	_	_	Heterozygous	a/a
MS-23	_	_	_	_	_	a/a
MS-24	_	_	_	_	Heterozygous	a/a
MS-25	_	_	_	Homozygous	_	a/a

Supplementary Table S4: Laboratory findings of patients with multiple sclerosis (MS) for thrombophilia risk factors

No	Factor V Leiden (R506Q)	Factor V R2 (H1299R)	Prothrombin (20210G>A)	Factor XIII V34L	beta-Fibrinogen -455 G>A	Human Platelet Antigen 1 (HPA-1) a/b
MS-26	_	_	_	Heterozygous	Homozygous	a/a
MS-27	_	_	_	_	_	a/a
MS-28	_	_	_	_	Homozygous	a/a
MS-29	_	_	_	Heterozygous	_	a/a
MS-30	_	_	_	_	Heterozygous	a/b
MS-31	_	_	_	_	Homozygous	a/a
MS-32	Heterozygous	_	_	_	Heterozygous	a/b
MS-33	_	_	_	_	_	a/b
MS-34	_	_	_	_	_	a/b
MS-35	_	Heterozygous	_	_	Heterozygous	a/b
MS-36	_	_	_	_	_	a/a
MS-37	Heterozygous	Heterozygous	_	_	Heterozygous	a/a
MS-38	Heterozygous	_	_	Heterozygous	_	a/a
MS-39	_	Heterozygous	_	_	Heterozygous	a/a
MS-40	_	_	_	_	Heterozygous	a/a
MS-41	_	_	_	_	_	a/b
MS-42	_	Heterozygous	Heterozygous	_	_	a/a
MS-43	Heterozygous	Heterozygous	_	Homozygous	_	a/a
MS-44	_	_	_	Heterozygous	Homozygous	a/a
MS-45	Heterozygous	_	_	_	Heterozygous	a/a
MS-46	_	_	_	Heterozygous	_	a/b
MS-47	_	_	_	Heterozygous	Heterozygous	a/a
MS-48	_	_	_	Heterozygous	_	a/b

Supplementary Table S5: Laboratory findings of patients with multiple sclerosis (MS) for cardiovascular risk factors

**No**	**Plasminogen activator inhibitor 1 (PAI-1) 4G/5G**	**Methylenetetrahydrofolate reductase (MTHFR) 677C>T**	**Methylenetetrahydrofolate reductase (MTHFR) 1298A>C**	**Angiotensin-Converting Enzyme (ACE) I/D**	**Apolipoprotein B (Apo) B R3500Q**
MS-1	4G/5G	Heterozygous	Heterozygous	I/D	_
MS-2	5G/5G	Heterozygous	_	D/D	_
MS-3	4G/4G	Heterozygous	_	D/D	_
MS-4	4G/5G	Homozygous	_	D/D	_
MS-5	5G/5G	Heterozygous	Heterozygous	D/D	_
MS-6	4G/5G	Heterozygous	_	D/D	_
MS-7	4G/5G	Heterozygous	_	I/D	_
MS-8	4G/5G	Heterozygous	_	D/D	_
MS-9	5G/5G	Heterozygous	_	I/D	_
MS-10	4G/5G	_	_	I/D	_
MS-11	5G/5G	Heterozygous	_	I/D	_
MS-12	4G/5G	_	_	I/D	_
MS-13	4G/5G	Heterozygous	_	I/D	_
MS-14	4G/4G	_	Homozygous	I/D	_
MS-15	5G/5G	_	Homozygous	D/D	_
MS-16	4G/4G	Heterozygous	Heterozygous	I/D	_
MS-17	5G/5G	_	Heterozygous	D/D	_
MS-18	4G/5G	Heterozygous	Heterozygous	I/D	_
MS-19	4G/5G	_	Heterozygous	I/D	_
MS-20	5G/5G	Heterozygous	Heterozygous	I/I	_
MS-21	4G/4G	Heterozygous	_	I/D	_
MS-22	4G/4G	Heterozygous	Heterozygous	D/D	_
MS-23	4G/5G	Heterozygous	Heterozygous	I/D	_
MS-24	5G/5G	Heterozygous	Heterozygous	I/D	_
MS-25	4G/5G	Homozygous	_	D/D	_

Supplementary Table S5: Laboratory findings of patients with multiple sclerosis (MS) for cardiovascular risk factors

No	Plasminogen activator inhibitor 1 (PAI-1) 4G/5G	Methylenetetrahydrofolate reductase (MTHFR) 677C>T	Methylenetetrahydrofolate reductase (MTHFR) 1298A>C	Angiotensin-Converting Enzyme (ACE) I/D	Apolipoprotein B (Apo) B R3500Q
MS-26	5G/5G	_	Heterozygous	I/D	_
MS-27	4G/5G	Heterozygous	_	I/D	_
MS-28	4G/5G	_	Heterozygous	I/I	_
MS-29	5G/5G	Heterozygous	Heterozygous	D/D	_
MS-30	5G/5G	_	_	D/D	_
MS-31	4G/4G	Heterozygous	Heterozygous	I/D	_
MS-32	5G/5G	_	_	I/D	_
MS-33	4G/5G	_	_	I/D	_
MS-34	4G/5G	Homozygous	_	D/D	_
MS-35	5G/5G	_	Heterozygous	D/D	_
MS-36	4G/4G	_	Heterozygous	I/D	_
MS-37	5G/5G	_	Heterozygous	D/D	_
MS-38	5G/5G	Heterozygous	Heterozygous	I/I	_
MS-39	5G/5G	Heterozygous	Heterozygous	I/D	_
MS-40	4G/4G	Heterozygous	Heterozygous	D/D	_
MS-41	5G/5G	Heterozygous	Heterozygous	I/D	_
MS-42	4G/5G	_	Homozygous	I/D	_
MS-43	4G/5G	Homozygous	_	I/D	_
MS-44	4G/5G	Homozygous	_	I/D	_
MS-45	5G/5G	_	Heterozygous	D/D	_
MS-46	4G/5G	_	Homozygous	I/D	_
MS-47	5G/5G	Heterozygous	_	D/D	_
MS-48	4G/5G	_	Heterozygous	D/D	_
